# IDO-induced immunosuppressive tryptophan catabolism following primary HIV infection

**DOI:** 10.1186/1471-2334-14-S2-O14

**Published:** 2014-05-23

**Authors:** Mohammad-Ali Jenabian, Kishanda Vyboh, Ido Kema, Cynthia Kanagaratham, Danuta Radzioch, Norbert Gilmore, Petronela Ancuta, Cécile Tremblay, Jean-Pierre Routy

**Affiliations:** 1 Chronic Viral Illnesses Service of the McGill University Health Center, Montreal, Canada; 2 Research Institute of the McGill University Health Center, Montreal, Canada; 3 Department of Laboratory Medicine, University Medical Center, Groningen, University of Groningen, The Netherlands; 4 University of Montreal, Department of microbiology and immunology, Montreal, Canada; 5 CHUM Research Center, Montreal, Canada

## Background

We showed in cross-sectional studies that tryptophan (Trp) catabolism into kynurenine (Kyn) by IDO enzyme expressed by dendritic cells (DC) contributes to regulatory T-cells (Tregs) expansion and immune suppression in chronic HIV infection. We prospectively assessed Trp catabolism and anti-inflammatory response following primary HIV infection (PHI).

## Methods

Plasma and Peripheral blood mononuclear cells (PBMCs) were longitudinally collected in 41 PHI patients (infection <90 days), 24 remained untreated (ART-naive) and 17 were ART-treated one year later. In addition, samples from elite controllers (EC, n=12) and healthy subjects (HS, n=12) were also assessed. IDO enzymatic activity marker (Kyn/Trp ratio) was measured by isotope dilution tandem mass spectrometry. IL-6, IL-18, TNF-α and IP-10 plasma levels were assessed by Luminex. Frequency of Tregs (CD4+CD25highCD127lowFOXP3high), CD11c+ myeloid DC (mDC) and CD123+ plasmacytoid DC (pDC) as well as HLA-DR/CD38 co-expression of on T-cells were assessed.

## Results

PHI patients had elevated Kyn/Trp ratio compared to HS and EC and further increased during the chronic phase, while normalized following ART. Accordingly, an increase of Treg frequency was observed at the baseline and continues to increase in the chronic phase only for those remaining untreated, when compared to HS and EC. Conversely, the frequency of mDC and pDC decreases over time only for those who remained untreated. Higher Kyn/Trp ratios were inversely correlated with the frequency of mDC and pDC at PHI and for those untreated. Importantly, the highest level of immune activation (HLA-DR+CD38+ CD8 T-cells) was observed during PHI followed by a decrease in chronic phase in ART-naïve and became comparable to EC and HS when receiving ART. Importantly, Kyn/Trp ratio was correlated with level of CD8 T-cell activation during PHI and for those who remained untreated. In line with this, positive correlations were observed between Kyn/Trp ratio and levels of IL-18 and TNF-α as well as markers of HIV disease progression IL-6 and IP-10.

## Conclusion

The progressive increase of Kyn/Trp ratio observed in the chronic phase of HIV infection in contrast to decreased viral load and T-cell activation, support the contribution of tissue damage and/or myeloid inflammatory syndrome in addition to viral replication for the development of immunosuppression.

